# Supplementary figures and images for: Parkinson’s disease-linked Parkin mutations impair glutamatergic signaling in hippocampal neurons
Source: BMC Biol. 2018 Sep 10;16:100. doi: 10.1186/s12915-018-0567-7 (PMC6130078; doi:10.1186/s12915-018-0567-7)

**Figure S1**

**a**

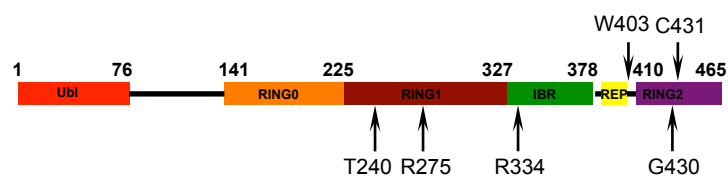

**b**

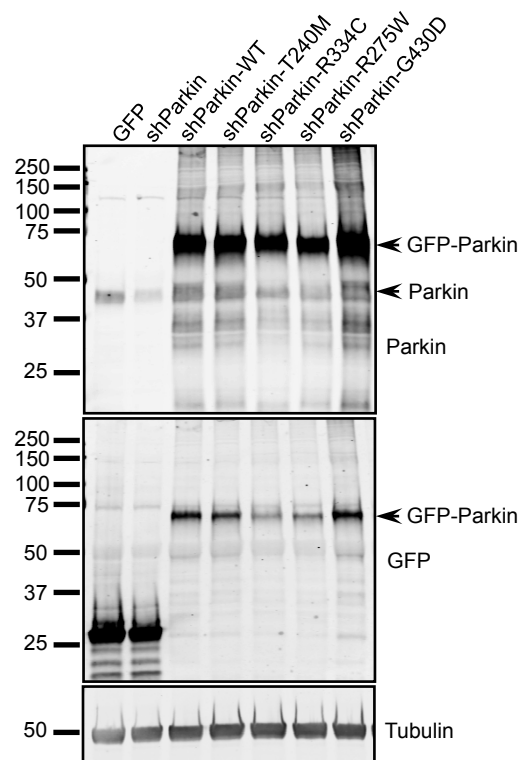

**c**

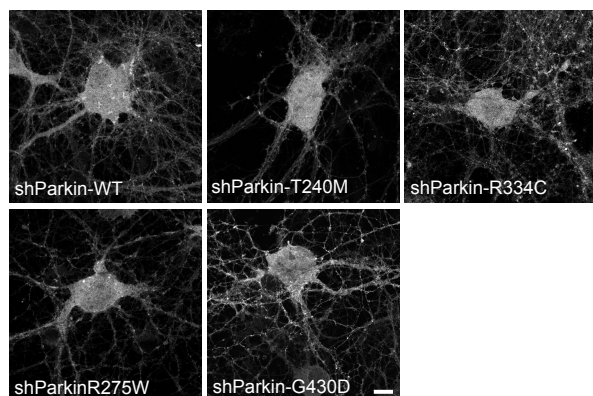

**d**

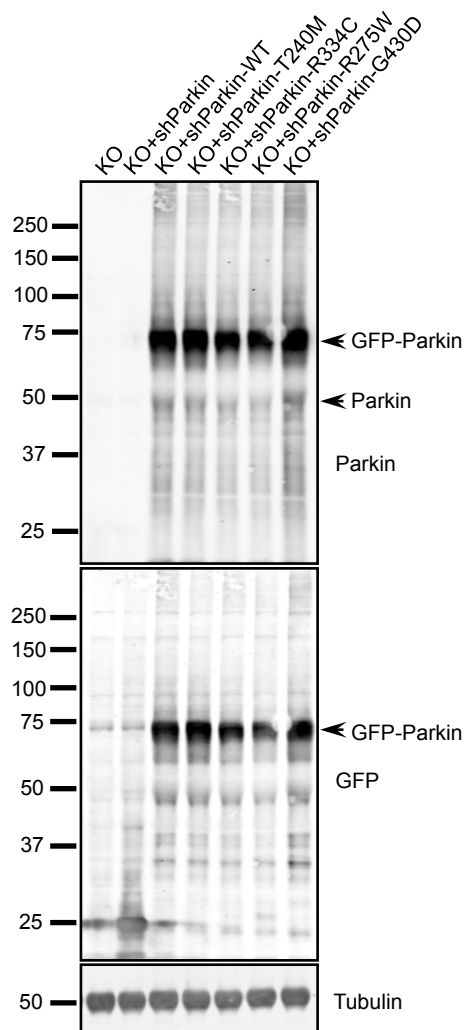

**e**

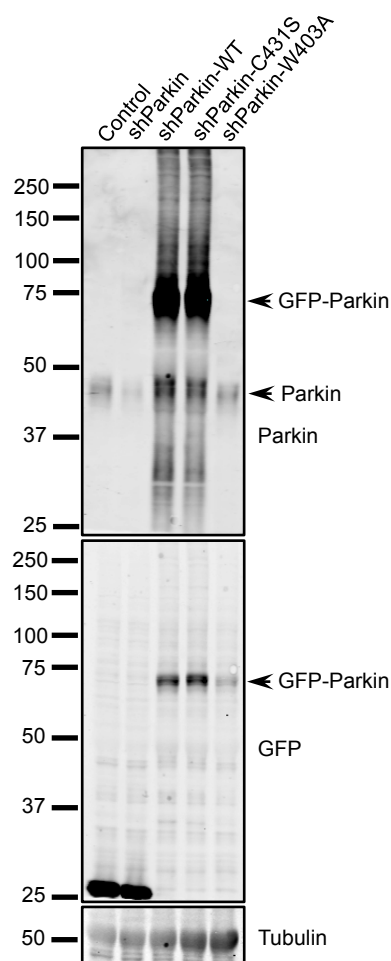

Supplement: Supplementary file 1 — Figure S1. Expression of Parkin mutants in hippocampal neurons. (a) Schematic diagram of Parkin showing domains and mutation/deletion sites. (b) Immunoblots of lysates from 14 DIV hippocampal neurons expressing GFP control, shParkin, shParkin-WT, shParkin-T240M, shParkin-R275W, shParkin-R334C, or shParkin-G430D constructs, probed for Parkin, GFP, and tubulin. (c) Representative images of shParkin-WT, shParkin-T240M, shParkin-R334C, shParkin-R275W and shParkin-G430D expression in 14 DIV hippocampal neurons. Scale bar, 10 μm. (d) Immunoblots of lysates from 14 DIV Parkin KO hippocampal neurons expressing shParkin, shParkin-WT, shParkin-T240M, shParkin-R275W, shParkin-R334C or shParkin-G430D constructs and Parkin KO control, probed for Parkin, GFP, and tubulin. (e) Immunoblots of lysates from hippocampal neurons expressing GFP, shParkin, shParkin-WT, shParkin-C431S, or shParkin-W403A, probed for Parkin, GFP, and tubulin. Note that GFP-Parkin W403A is not recognized by the Parkin antibody but is detected by the GFP antibody. (PDF 3931 kb) [file 12915_2018_567_MOESM1_ESM.pdf]

Figure S2

a

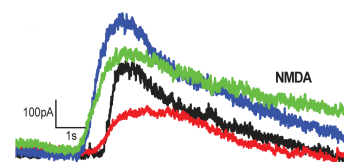

NMDA Peak Current

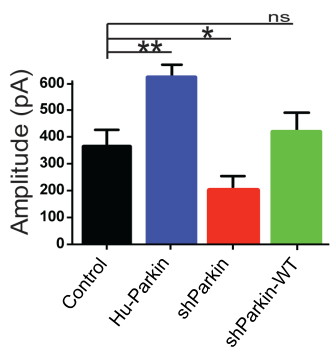

b

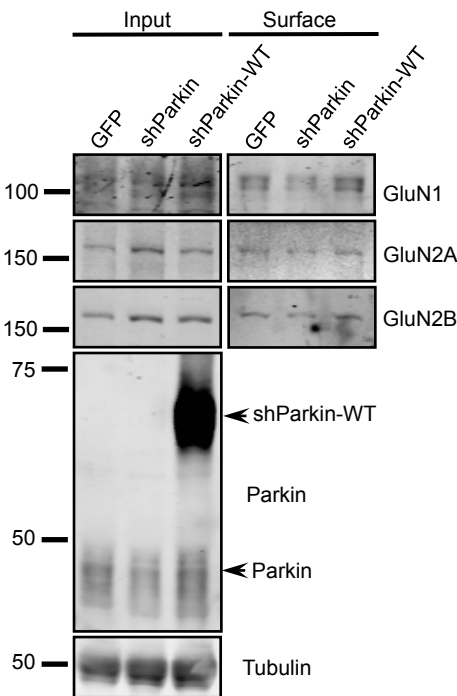

c

Surface/Input GluN

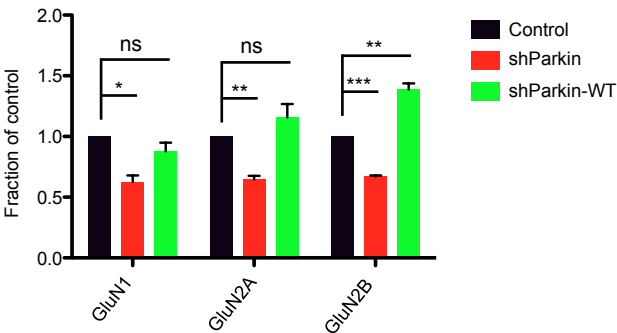

Supplement: Supplementary file 2 — Figure S2. Parkin deficiency leads to reduced NMDAR-mediated currents and cell-surface levels. (a) Representative traces of whole-cell currents (upper panel) induced by local application of 100 μM NMDA to hippocampal neurons expressing GFP, human Parkin (Hu-Parkin), shParkin, or shParkin-WT constructs, and quantification of peak current amplitudes induced by NMDAR activation for these conditions (lower panel) (n = 19 for control, 24 for shParkin, 22 for hu-Parkin, 14 for rescue; *P < 0.05, **P < 0.005, one-way ANOVA, error bars represent SEM). (b) Representative immunoblots of total input (left) and surface biotin-labeled fractions (right) from 14 DIV hippocampal neuron lysates expressing GFP, shParkin or shParkin-WT constructs. (c) Quantification of surface/input ratio, normalized to GFP control condition and expressed as a fraction of control. (n = 3 experiments; *P < 0.05; **P < 0.01, ***P < 0.001, unpaired t test, error bars represent SEM). (PDF 215 kb) [file 12915_2018_567_MOESM2_ESM.pdf]

Figure S3

a

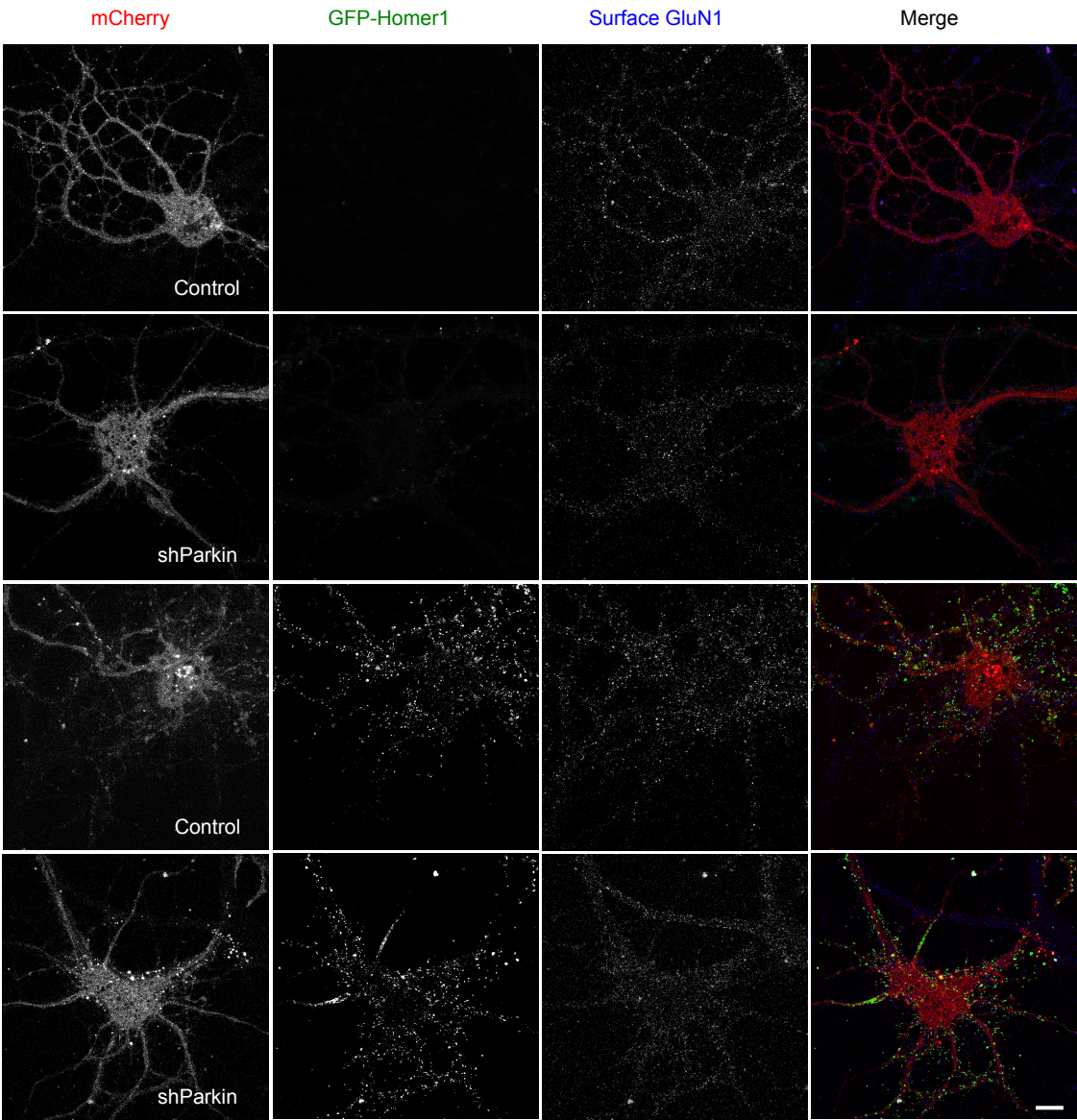

b

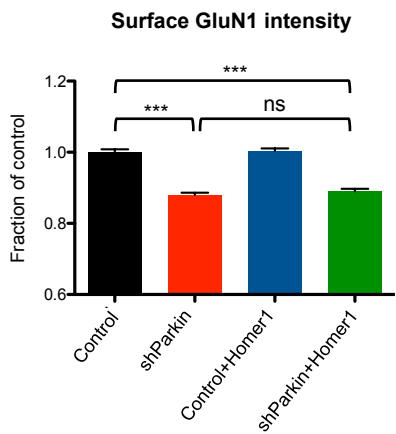

Supplement: Supplementary file 3 — Figure S3. Homer1 overexpression does not rescue cell-surface NMDAR levels in Parkin knockdown neurons. (a) Representative images of hippocampal neurons co-transfected from 6 to 14 DIV with mCherry +/− shParkin alone or with GFP-Homer1, and immunostained for surface GluN1. Scale bar, 10 μm. (b) Quantification of cell surface GluN1, expressed as a fraction of mCherry control (n ≥ 40 fields of view per condition with > 100 GluN1 puncta per field, results confirmed in 3 independent experiments. ***P < 0.001, one-way ANOVA, error bars represent SEM). (PDF 5670 kb) [file 12915_2018_567_MOESM3_ESM.pdf]

Figure S4

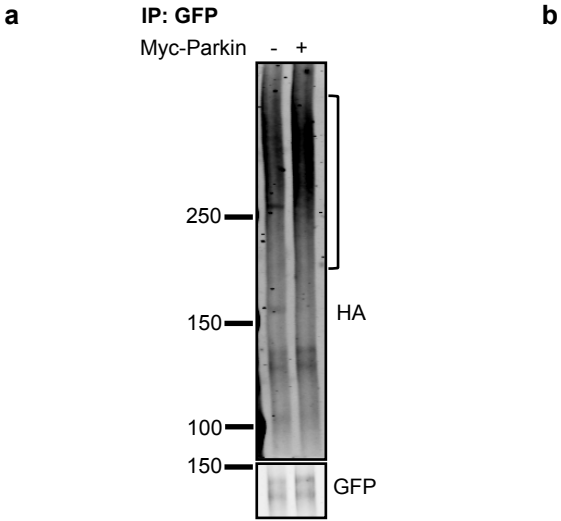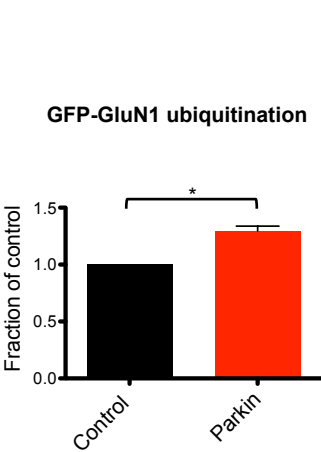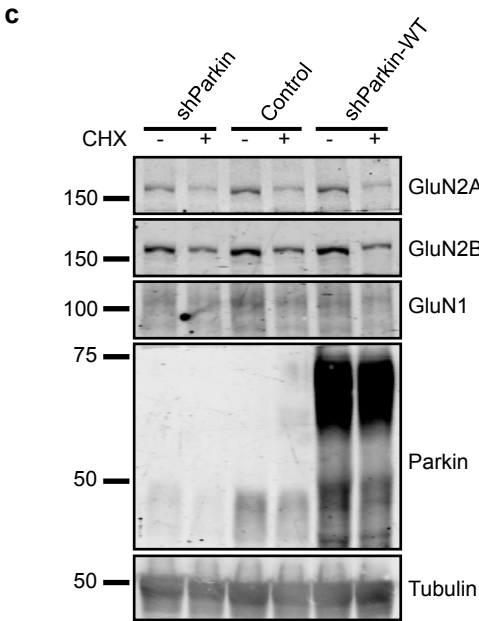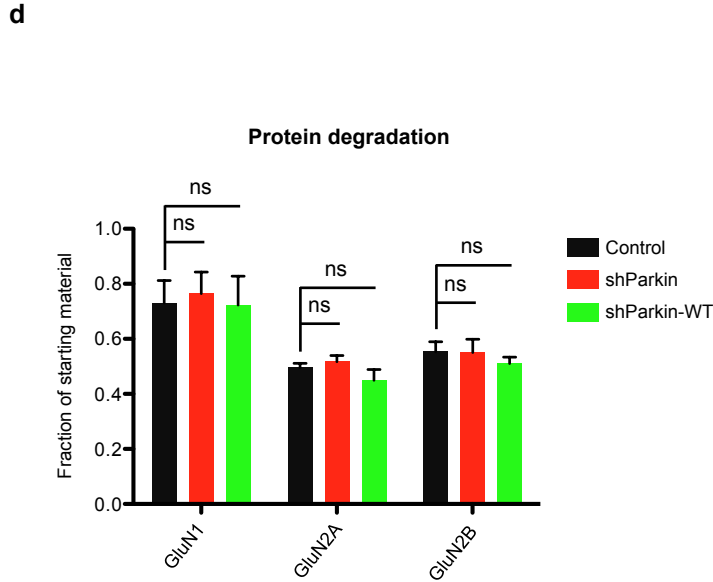

Supplement: Supplementary file 4 — Figure S4. Parkin-mediated ubiquitination does not induce NMDAR degradation. (a) Representative immunoblots for GFP immunoprecipitation (IP) under denaturing conditions (1% SDS) from HEK293T cell lysates expressing Myc/Myc-Parkin, GFP-GluN1 and HA-ubiquitin, probed for HA and GFP. Ubiquitin immunoreactivity used for quantification is marked on HA blots. (b) Quantification of GFP-GluN1 ubiquitination by measurement of marked HA blot intensity, normalized to immunoprecipitated GFP-GluN1 and reported as a fraction of Myc control. (n = 3 experiments, *P < 0.05; one-way ANOVA, error bars represent SEM). (c) Representative immunoblots of lysates from 14 DIV hippocampal neurons expressing GFP/shParkin/shParkin-WT constructs, treated with DMSO vehicle control (−) or cycloheximide (CHX) and probed with the indicated antibodies. (d) Quantification of protein intensity after 24 h of CHX treatment, normalized to tubulin and reported as a fraction of the DMSO control intensity (starting material) (n = 3 experiments, unpaired t test, error bars represent SEM). (PDF 178 kb) [file 12915_2018_567_MOESM4_ESM.pdf]

Figure S5

**a**

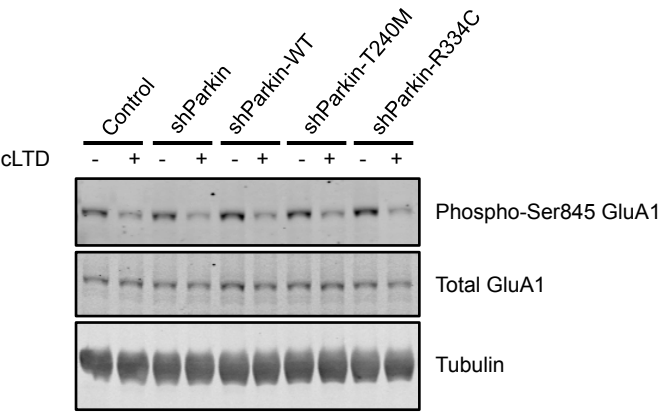

**b**

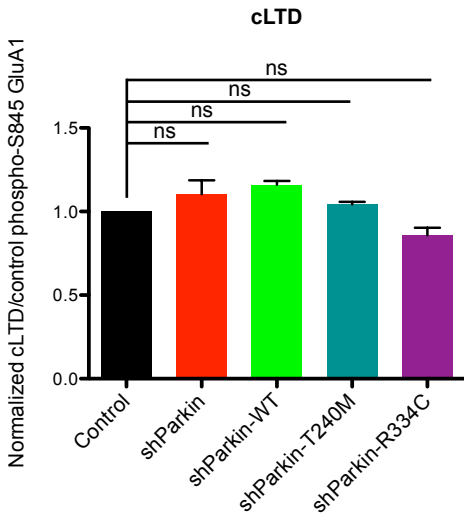

Supplement: Supplementary file 5 — Figure S5. Parkin has no effect on phospho-Serine 845 GluA1 reduction during cLTD. (a) Representative immunoblots of lysates from 14 DIV hippocampal neurons expressing GFP/shParkin/shParkin-WT/-T240M/-R334C constructs, under control condition (−) or after cLTD induction (+) and probed with the indicated antibodies. (b) Quantification of the ratio of phospho-Serine 845 GluA1 intensity with cLTD to control condition, normalized to GFP control. (n = 3 experiments, one-way ANOVA, error bars represent SEM). (PDF 119 kb) [file 12915_2018_567_MOESM5_ESM.pdf]
